# Differential Impact of Social and Non-social Cues on Decision-making Across At-risk Mental State, Early Psychosis, and Healthy Participants

**DOI:** 10.1093/schizbullopen/sgag005

**Published:** 2026-02-26

**Authors:** Juliet Denise Griffin, Alexandrina Vasilichi, Joost Haarsma, Eleonore van Sprang, NSPN Consortium, Edward Bullmore, Raymond Dolan, Ian Goodyer, Peter Fonagy, Peter Jones, Samuel Chamberlain, Michael Moutoussis, Tobias Hauser, Sharon Neufeld, Rafael Romero-Garcia, Michelle St Clair, Petra Vértes, Kirstie Whitaker, Becky Inkster, Gita Prabhu, Cinly Ooi, Umar Toseeb, Barry Widmer, Junaid Bhatti, Laura Villis, Ayesha Alrumaithi, Sarah Birt, Aislinn Bowler, Kalia Cleridou, Hina Dadabhoy, Emma Davies, Ashlyn Firkins, Sian Granville, Elizabeth Harding, Alexandra Hopkins, Daniel Isaacs, Janchai King, Danae Kokorikou, Christina Maurice, Cleo McIntosh, Jessica Memarzia, Harriet Mills, Ciara O’Donnell, Sara Pantaleone, Jenny Scott, Beatrice Kiddle, Ela Polek, Pasco Fearon, John Suckling, Anne-Laura van Harmelen, Rogier Kievit, Richard Bethlehem, Graham K Murray, Paul Charles Fletcher

**Affiliations:** Computational Psychiatry Research Group, Department of Psychiatry, University of Oxford, Oxford, OX3 7JX, United Kingdom; Department of Computational Neuroscience, Max Planck Institute for Biological Cybernetics, Tübingen, 72076, Germany; Department for Computational Neuroscience, University of Tübingen, Tübingen, 72076, Germany; Faculty of Psychology and Neuroscience, Maastricht University, Maastricht, 6229 ER, Netherlands; De Jeugdautoriteit, Utrecht, 3584 BX, Netherlands; Cambridgeshire and Peterborough NHS Trust, Cambridge, CB4 1PX, United Kingdom; Department of Psychiatry, University of Cambridge, Cambridge, CB2 8AH, United Kingdom; Cambridgeshire and Peterborough NHS Trust, Cambridge, CB4 1PX, United Kingdom; Department of Psychiatry, University of Cambridge, Cambridge, CB2 8AH, United Kingdom; Wellcome Trust MRC Institute of Metabolic Science

**Keywords:** delusions, predictive processing, decision-making under uncertainty

## Abstract

**Background and Hypothesis:**

Clinical observations of psychotic experiences reveal a predominance of social themes, raising an important question about whether the underlying information-processing alterations show an analogous domain-specificity particularly impacting social inferences, including beliefs about others’ intentions.

**Study Design:**

We examined how learning and decision-making in relation to an external cue was influenced by whether its source was presented as social or non-social, in healthy controls (HCs) compared to people with first episode psychosis (FEP) or with an at-risk mental state (ARMS). Using computational modeling, we quantified how responses were biased by the external cue, and were differently sensitive to its utility, depending on its being perceived as social versus non-social in origin.

**Study Results:**

Overall, tendency toward choosing the externally-cued option was lower in HCs than both clinical groups, and higher in FEP than ARMS. In HCs, there was a stronger bias toward the external cue when it was presented as social in origin: in contrast, the bias was unaffected in the ARMS group, and the FEP group conversely showed relatively greater bias toward the non-social cue. The effect of the cue’s perceived origin on sensitivity to its objective value likewise differed between groups: despite being more biased toward the social cue, HCs were relatively less able to use social information to optimize their choices, whereas sensitivity to the cue’s current value was unaffected by social/non-social origin in ARMS and FEP.

**Conclusions:**

Our findings contradict ideas that psychosis is associated with a simple and specific deficit in social processing.

## Introduction

Any satisfactory theory of psychosis must account for positive symptoms’ “fundamentally social nature,”^1^ including the striking predominance of social themes in their semantic content. Paranoid delusions are the most common form transdiagnostically: schizophrenia-related, Parkinsonian, and drug-induced delusions all tend to center around persecution,^2^ as do “quasi-psychotic” thoughts and preoccupations in borderline personality disorder.^3,4^ While delusional themes are not necessarily fixed across phases or episodes of psychotic illness,^5^ the typical pattern of thematic content across clinically-presenting delusions remains largely stable across successive patient cohorts,^6^ with a consistent preponderance of social themes. Although delusions’ precise content reflects preoccupations of contemporary culture as well as factors particularly salient to the affected individual, the persecution theme itself as a central motif of delusional thought is remarkably culturally invariant.^7–9^

Other transdiagnostically-prevalent delusions concerning social or relational phenomena include misidentification, erotomania, delusional jealousy, and ideas of reference. It is noteworthy too that, while delusions of grandiosity, religiosity, and/or guilt^10^ are not inherently social in their defining content, they commonly present with a social theme^11^ and involve other agents (human or spiritual). It might even be argued that the less obviously social delusions of alien control, thought insertion, and thought broadcasting also implicitly or explicitly invoke an external agent.^1^ Moreover, one important formal characteristic, distinguishing delusions in general from beliefs that are odd, entrenched, and/or false but nevertheless non-clinical, is a characteristic “Global social estrangement…[and] failure to treat others as reliable sources of information.”^12^

A theoretical account of delusions must therefore include their tendency to revolve around social relations and other agents. Some have argued that a predictive processing account of psychosis is too general to explain delusions’ essentially social nature.^13^ One possible response has been to consider the incorporation of domain-specificity into the predictive processing framework^14–16^ despite the fact that most formulations of predictive processing are domain-general.^17–19^ Another possibility is that we need not invoke such mechanistic domain-specificity to account for delusions’ social content because social themes and events are in themselves more computationally complex and uncertain^20,21^ meaning that domain-general predictive processing perturbations will most readily and obviously manifest in such themes.^22^

So the question arises: is psychosis associated with particular alterations specifically in the processing of information that is social in its content or origin?^23–26^ While there are well-documented domain-general alterations to inferences under uncertainty,^27,28^ whether psychosis more particularly affects inferences in the social domain remains hotly debated.^29–31^ The current study sought to address this, using a non-competitive observational learning and decision-making task to investigate whether and how social information is treated differently from non-social information, in healthy controls (HCs) compared to people with first episode psychosis (FEP) and people with an at-risk mental state (ARMS). Our task was based on a previous study which independently manipulated the reliability of information and its perceived source (social or non-social).^32^ In brief, a participant is required to choose between 2 stimuli that are probabilistically rewarded. Their decision can be based both on their own experience acquired over the course of multiple trials in which they learn which stimulus is more likely to be rewarded, and on an external cue or “hint” in the form of a red frame around one of the stimuli. They are led to believe that this hint was either based on prior choices of other participants (social) or was computer-generated (non-social). Using a within-subjects manipulation, we assessed the degree to which HC, ARMS, and FEP participants differed in bias toward the external cue as a function of whether its origin was perceived to be social or non-social. We then explored whether sensitivity to objective differences in the utility of external information was moderated by its perceived source differently across these 3 groups.

## Methods

### Sample

Participants were 14-35 years old, with no history of neurological disorders/trauma and no learning disability requiring specialist educational support/treatment. All participants understood written and spoken English and gave informed consent.

Participants with an ARMS (*n* = 12) were recruited through Cambridgeshire and Peterborough’s early onset psychosis service (CAMEO) and via direct contact with individuals who, having previously participated in unrelated Neuroscience in Psychiatry Network (NSPN) studies, had records indicating willingness to be invited for future experiments and membership in a high-schizotypal, low-mood, help-seeking sub-group identified by latent sub-class analysis.^33^ NSPN cohort members who, at last contact, were help-seeking and had upper-quartile scores on the Mood and Feelings Questionnaire (MFQ)^34^ and the Schizotypal Personality Questionnaire^35^ were invited for an initial screening assessment, involving a structured clinical interview^36^ to determine eligibility for ARMS group inclusion. Participants recruited into the ARMS group through CAMEO had all during the past 6 months met clinical criteria for ARMS. At the time of testing, all ARMS participants were confirmed to meet ARMS criteria based on attenuated psychotic symptoms thresholds, as measured by the Comprehensive Assessment for At Risk Mental States (CAARMS),^36^ and none had ever experienced a full-blown psychotic episode.

Participants in the FEP group (*n* = 14) were recruited through CAMEO, having been diagnosed with a psychotic episode, for the first time, within the previous 5 years. Structured clinical interviews by researchers confirmed that, at time of testing, each FEP participant had current symptoms meeting the CAARMS threshold for full-blown psychosis.^36^

Matched HCs (*n* = 14) were recruited from the NSPN database. Individuals invited to participate in the HC group, at last contact, had expressed interest in future participation and had lower-quartile MFQ scores. Structured clinical interviews confirmed no HC participant had any indication of past or present mental illness. For sample demographics and clinical characteristics, see Supplementary material (Tables S1-S2).

**Figure 1:**
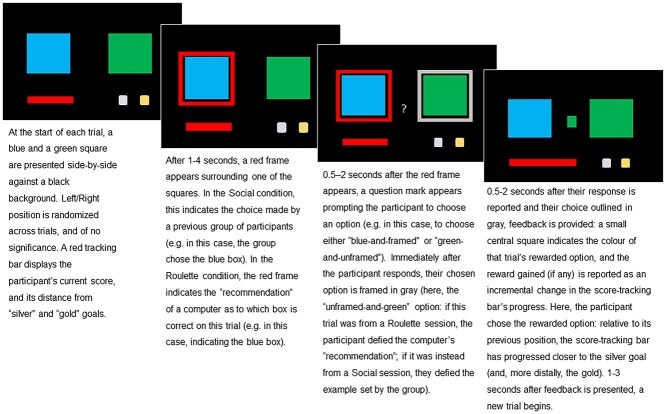
Schematic Illustration of a Single Trial.

### Task

Each participant performed 2 separate 120-trial sessions of a task in which the values of 2 independent cues were simultaneously learned from ambiguous feedback, and deployed trialwise to estimate the overall expected values of 2 choice options uniquely defined by those cues.^32^ The cues were: the option’s *color (blue or green)*, and whether or not it was surrounded by a *red frame* (Figure 1).

Option color was a “direct cue” whose significance could only be learned from personal experience of reward history within a session. The red frame was an “indirect cue” in that it represented a “hint” derived from an external source, which the participant could either follow (by choosing the framed option) or defy (by choosing the unframed option). Note that the predictive value of this hint could be continuously updated throughout the session in the same iterative, trialwise manner as that of the “direct cue.” To manipulate the perceived source of this hint between sessions, participants were told either that the red frame was an indication provided as output from the computer, or that it reflected previous choices made by a group of other participants. The instructions used to effect this manipulation were those used in previous between-subjects studies deploying this task to investigate social versus non-social learning in healthy participants.^32,37,38^ By manipulating instructed beliefs about the external cue’s source across sessions, and using computational modeling to quantify the objective predictive significance that could be estimated for a cue on each trial, we could investigate how equivalent information from a perceptually-identical external cue (the red frame) was learnt and weighted differently when presented as originating from a social versus a non-social source. This allowed us to assess group differences in the tendency to accept and use external cues in decision-making, and to determine whether such information’s perceived source (social or non-social) impacted how it was learnt about and weighed differently between HC, ARMS, and FEP.

### Social/Non-social Condition Manipulation

To manipulate perceived source of the indirect information, immediately before each of the 2 sessions participants received a different set of verbal and visually-presented instructions about the origin of this external cue. These instructions were the same as the “Social” and “Roulette” task instructions that previous work using this paradigm deployed to achieve the required social/non-social manipulation (see Supplementary Material).^32,37,38^ Which of the 2 sessions was undertaken first was pseudorandomized to ensure parity between groups, and to ensure neither condition was more frequently undertaken before the other across participants. The 2 sessions were administered on separate days, to reduce the possibility that practice effects might alter performance in the second session.

Before the Social Condition session, participants were instructed that the red frame reflected choices made by a previous group of players on that same task, but that this group’s trialwise choices (indicated by the red frame) had been “juggled” such that “in some phases, they won’t seem very useful—for example, they could be guesses from the very beginning of the task when they had little experience. In other phases, however, they will seem quite useful—for example, responses from later in the task when they had had the opportunity to practice a bit more.”^32^ Before the Non-Social (or “Roulette”) Condition session, participants were instructed that the red frame was a “suggestion” generated by a computerized roulette wheel: “on each trial the computer spins the roulette, if the ball lands on black the computer will put a frame around the correct answer, if the ball lands on red the computer will frame the incorrect answer. The only catch is that there are different types of roulette wheel. Some roulette wheels are half red and half black. This type of roulette is equally likely to give you correct and incorrect suggestions. However, others are biased. This type of roulette will give you either mostly correct or mostly incorrect suggestions. Once the computer has selected a roulette wheel it will stick with that wheel for a while. However, it will switch between the various different roulette wheels throughout the course of the experiment.”^32^ The notion of the computerized roulette wheel was introduced as a visual analogy to express the important point that, unlike most computers (normally programmed to consistently produce the “right” answer), and unlike most roulette wheels (normally constructed with a fixed and equal number of black and red segments to ensure 50% probability of landing on the “right” answer) the process generating the computerized “hint” was probabilistic and subject to change across phases of the session. That subjects in this study, as in previous healthy volunteer studies validating this approach, understood the roulette metaphor as intended (rather than mistakenly equating the red frame with an ordinary roulette wheel whose output would be random and therefore uninformative), is evident from the finding that all 3 groups successfully learnt and weighted information about the red frame’s objective predictive value across both sessions.

### Analysis

Overall accuracy, and overall proportion of red-framed (RF) choices, were calculated for Social and Roulette Condition sessions for each subject. Preliminary behavioral analysis of these metrics in relation to experimental factors of interest (Group, Condition, and their interaction) could not unambiguously address the key scientific questions of whether and how learning and decision-making were differently affected by the external cue as a function of its perceived source across groups. Two subjects may show similar overall accuracy while relying on different combinations of available information: while sensitivity to the epistemic value of the external red-frame cue is one factor that contributes to overall accuracy, sensitivity to the value of an option based on individual trial-and-error learning about its color contributes also. Likewise, there are various reasons the red-framed (RF) square may be chosen (or forgone) on any given trial, rendering average proportion of RF choices within a session ambiguous. A participant’s tendency to choose the red-framed option cannot be straightforwardly interpreted as a bias, because it is conflated with their sensitivity to that option’s current overall estimated value. In turn, a subject’s sensitivity to the differential expected value of the 2 options reflects not only their learnt estimate about the predictive value of the red frame, but also what they have learnt about the value of the direct cue (ie, an option’s color).

Computational modeling was therefore required to parse out the contributions of (1) direct information learned from experience of the predictive value of option color and (2) indirect information learned from the observed association between the external RF cue and reward—both from one another, and from (3) variations in the tendency to accept or reject (conform with or depart from) the indication of the RF cue depending on whether its source is perceived as social or non-social. We obtained independent trialwise expected values of the direct cue (ie, blue relative to green) and the indirect cue (ie, red-framed relative to unframed) that a Bayes-optimal learner exclusively tracking that domain would estimate, given the sequence of previous inputs within that session.^32^ Rather than estimating coefficients for subjects’ correspondence to these Bayesian-Learner estimates in isolation from one another (as did previous between-subjects research using Social and Roulette versions of this task)^32^ we capitalized on our within-subjects experimental design by estimating correspondence to these Bayesian-ideal observers as a linear function of fixed (population and group) and random (subject-specific) effects, inferring all effect size estimates simultaneously within a single general linear model (GLM) fitted to the entire dataset using Maximum Pseudo-Likelihood estimation. ANOVA on the fixed effect terms of the best-fitting computational model was conducted to test the experimental hypotheses, and pairwise comparisons exploring the nature significant effects conducted using MATLAB’s coefTest function.

Finally, findings from this computational analysis were visualized in simulations illustrating how bias toward the external cue, and sensitivity to indirect and direct information, differed between conditions in each group.

**Figure 2:**
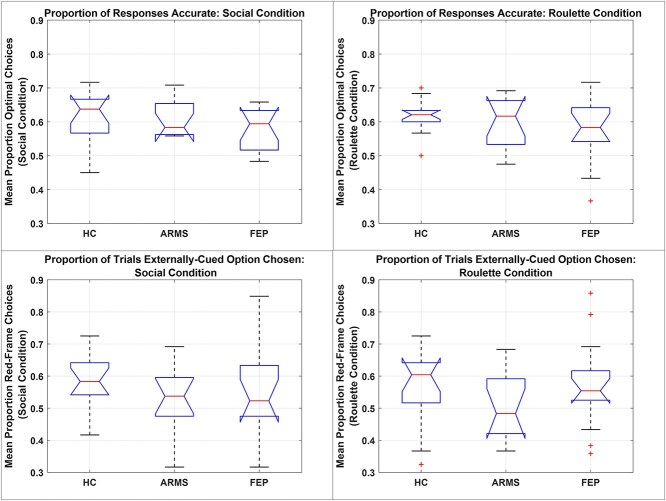
HC, ARMS, and FEP Group Distributions of: Accuracy (Top Row) and Tendency to Accept the External Cue’s Hint (Bottom Row); in Social session (Left Column) and in Roulette Session (Right Column). Responses were “accurate” (vs inaccurate) if the chosen option had the higher (vs the lower) probability of reward relative to currently-prevailing experimental contingencies. Responses were “externally-cued” if the subject chose the square surrounded by the red-frame stimulus (vs chose the unframed square). On each plot, central bars indicate group average; boxes indicate group interquartile range; dotted lines indicate group range; and crosses indicate any outliers in the relevant behavioral measure.

## Results

### Behavioral Summary Statistics (Figure 2)

Each subject’s average accuracy was calculated for Social and Roulette sessions separately, and predicted as a linear function of 3 fixed factors: Group, Condition, and the Group**:**Condition interaction. A random effect of subject identity did not significantly improve model fit (theoretical likelihood ratio test: *P* = .062), and was consequently dropped without impacting results’ direction or significance. ANOVA demonstrated a significant intercept term (*F* = 6044.4; df = 1,74; *P* < .001^**^), around which none of the experimental factors significantly contributed to observed variance in overall accuracy [Group (*F* = 2.366; df = 2,74; *P* = .101); Condition (*F* = 0.0320; df = 1,74; *P* = .858); Group**:**Condition (*F* = 0.082; df = 2,74; *P* = .922)]. Note that accuracy on this task reflects a combination of several factors: sensitivity to direct information, sensitivity to indirect information, and the interaction between a subject’s bias and the prevailing cue-outcome contingency. However, the findings are presented here for completeness; and demonstrate that all groups were capable of performing the task, in both conditions, with no significant overall differences that would indicate misunderstanding of task demands, or ceiling effects in performance.

The proportion of each participant’s choices that were of the red-framed (RF), rather than the unframed, option was calculated separately for Social and Roulette sessions. This measure was predicted by a linear mixed model including 3 fixed factors (Group, Condition, and their interaction) and a random intercept term (included here as it significantly improved model fit: theoretical likelihood ratio test (*P* = .000330^*^)). ANOVA on this model’s fixed effects found a significant fixed intercept (*F* = 1277.7; df = 1,74; *P* < .001^**^), but average proportion RF choices did not depend on Group (*F* = 1.27; df = 2,74; *P* = .290), Condition (*F* = 0.391; df = 1,74; *P* = .534), nor their interaction (F = 0.629; df = 2,74; *P* = .536). However, given this measure’s inherent ambiguity, computational modeling (to control for the contributions of the red-framed option’s objective value, based on all relevant predictive information) is required to determine whether these factors differentially affect bias toward the RF.

### Computational Modeling

Because information about an option’s value is available from both the direct cue (blue/green color) and the indirect cue (presence/absence of frame), which in any instance could concur or conflict with one another, computational modeling was required to estimate the relative influences of these 2 cues in shaping participants’ responses. The aim of these analyses was to simultaneously estimate how participants treated these different cues, and how their treatment of the indirect cue was affected by whether it was perceived to come from a social or non-social source. Ultimately, the aim was to compare this influence across HC, ARMS, and FEP.

Following previous work,^32^ the “Individual Bayesian Learner” (IndivBL) was so-called because it tracked the value of the direct cue, an option’s color, which could only be considered indicative as a result of the subject’s personal experience of blue and green’s outcome histories within the session; the “Red-Frame Bayesian Learner” (RFBL) meanwhile tracked the value of the indirect or external cue by observing whether its indication was associated with an option’s being rewarded or unrewarded across previous trials of that session.

Equation (1) describes the GLM used to predict binomial response data ([1;0] = [chose red-framed (RF) option; chose Unframed option]) as a function of 4 fixed factors: categorical variables Group and Condition, and continuous computational variables “IndivBL” and “RFBL.” Trialwise probability of choosing the red-framed option was modeled as the linear combination of 4 fixed main effects (representing the effects of these predictors on the grand mean intercept), 5 two-way interactions (between Group and Condition, and between each of these categorical factors with the continuous variables IndivBL and RFBL), and 2 three-way interactions (Group:Condition:IndivBL and Group:Condition:RFBL). Reported results are from the Probit link function, which provided better numerical model fit than the Logit link function without affecting results’ direction or significance. Random effects terms for intercept, IndivBL, and RFBL (correlated or uncorrelated) were omitted because all resulted in poorer fit than the fixed-effects only model, without affecting results’ direction or significance (see Supplementary material Table S3).


1 \begin{eqnarray*}& \boldsymbol{RFchosen}\sim \mathbf{1}+\boldsymbol{Group}+\boldsymbol{Cond}+\boldsymbol{IndivBL}+\boldsymbol{RFBL}\notag\\&+\boldsymbol{Group}:\boldsymbol{Cond}+\boldsymbol{Group}:\boldsymbol{IndivBL}+\boldsymbol{Group}:\boldsymbol{RFBL}\notag\\\notag&+\boldsymbol{Cond}:\boldsymbol{IndivBL}+\boldsymbol{Cond}:\boldsymbol{RFBL}\\&+\boldsymbol{Group}:\boldsymbol{Cond}:\boldsymbol{IndivBL}+\boldsymbol{Group}:\boldsymbol{Cond}:\boldsymbol{RFBL} \end{eqnarray*}

General Linear Model used to predict binomial response data. “Condition” is abbreviated to “Cond” (eqn 1)

**Figure 3:**
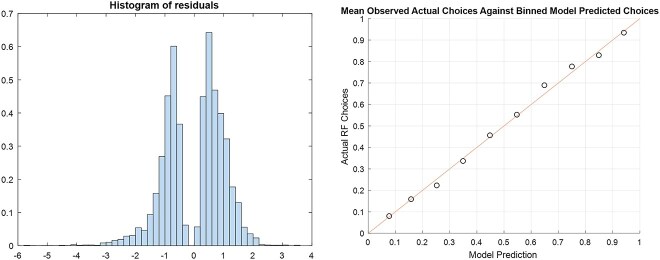
(A) Histogram of model residuals showing bimodal distribution centered around 0. (B) Scatter plot of average observed participant RF-choice data (y-axis), for each 0.1-sized “bin” of model- predicted P(choose RF) (*x*-axis) showing good correspondence between trialwise model predictions and actual participant choices across the full range of values.

The bimodal residual distribution has 2 reasonably symmetrical peaks centered around zero (Figure 3A) and the scatter plot of average predicted against actual values in each 0.1-sized “bin” lies near the 1:1 correspondence line (Figure 3B), indicating the model fits the data well, and provides a similarly good fit across the entire predicted range.^39^

**Table 1:** Fixed Effect Coefficients of General Linear Model (GLM) Predicting Trialwise RF Choices

Fixed effect coefficient	Est.	SE	*t*	df	*P*-value	95% CIs	
						Lower	Upper
**Intercept**	**−2.05**	**0.0527**	**−38.89**	**9580**	<.001^*^	**−2.15**	**−1.95**
Cond_SOCIAL	0.0027	0.0527	0.506	9580	.613	−0.077	0.130
**Group_HC**	**−0.545**	**0.0765**	**−7.179**	**9580**	<.001^*^	**−0.699**	**−0.399**
Group_ARMS	0.126	0.0751	1.680	9580	.0931	−0.021	0.273
**IndivBL**	**2.47**	**0.0735**	**33.64**	**9580**	<.001^*^	**2.33**	**2.62**
**RFBL**	**2.12**	**0.0728**	**29.13**	**9580**	<.001^*^	**1.98**	**2.26**
**Cond_SOCIALGroup_HC**	**0.274**	**0.0751**	**3.586**	**9580**	**.00033**	**0.124**	**0.424**
Cond_SOCIAL: Group_ARMS	−0.052	0.0765	−0.698	9580	.485	−0.110	0.285
Cond_SOCIAL: IndivBL	0.141	0.0735	1.914	9580	.0556	−0.0034	0.285
**Group_HC: IndivBL**	**0.773**	**0.106**	**7.326**	**9580**	<.001^*^	**0.566**	**0.980**
Group_ARMS: IndivBL	−0.188	0.105	−1.794	9580	.0729	−0.393	0.017
**Cond_SOCIAL: RFBL**	**−0.191**	**0.0728**	**−2.618**	**9580**	.0089^*^	**−0.333**	**−0.048**
**Group_HC: RFBL**	**0.557**	**0.105**	**5.311**	**9580**	<.001^*^	**0.351**	**0.763**
**Group_ARMS: RFBL**	**−0.313**	**0.104**	**−3.01**	**9580**	.0026^*^	**−0.517**	**−0.109**
Cond_SOCIAL: Group_HC: IndivBL	−0.158	0.105	−1.494	9580	.135	**−**0.365	0.0492
Cond_SOCIAL: Group_ARMS: IndivBL	0.013	0.105	0.123	9580	.902	−0.193	0.218
**Cond_SOCIAL: Group_HC:RFBL**	**−0.431**	**0.105**	**−4.107**	**9580**	<.001^*^	**−0.636**	**−0.225**
Cond_SOCIAL: Group_ARMS: RFBL	0.194	0.104	1.867	9580	.0619	−0.0097	0.398

Abbreviations: Est = estimate; *t* = t-statistic; Cond. = Condition; df = degrees of freedom. Significance (*P*-value) of each effect’s contribution to explaining variance around the grand mean (Intercept) is indicated. Significant fixed effects are printed in bold type-face, and an asterisk (*) symbol beside an effect's accompanying p-value indicates that it is significant (i.e. that *p* < 0.05).

ANOVA on the model coefficients in Table 1 demonstrated a significant intercept (*F* = 1512.6; df = 1,9580; *P* < .001^*^) around which variance in probability of choosing the red-framed option was explained by main effects of Group (*F* = 29.45; df = 2,9580; *P* < .001^*^), IndivBL (*F* = 1131.8; df = 1,9580, *P* < .001^*^), and RFBL (*F* = 848.79; df = 1,9580; *P* < .001^*^). There was no main effect of Condition (*F* = 0.256; df = 2,9580; *P* = .613), however, there was a significant Group:Condition interaction (*F* = 7.59; df = 2,9580; *P* = .000507^*^) indicating that bias toward the red frame depended upon its perceived external source differently across groups. In addition, there were significant 2-way interactions between IndivBL and Group (*F* = 30.02; df = 2,9580, *P* < .001^*^), between RFBL and Group (*F* = 14.11; df = 2,9580; *P* < .001^*^), and between Condition and RFBL (*F* = 6.86; df = 1,9580; *P* = .00885^*^) though importantly this latter interaction depended on group membership (ie, there was a significant 3-way interaction between Condition, Group, and RFBL: *F* = 8.521; df = 2,9580; *P* = .0002^*^). There was no significant interaction between Group, Condition, and IndivBL (*F* = 8.521; df = 2,9580; *P* = .237).

Group’s significant effect on the intercept reflected significant pairwise differences between HC and ARMS (*P* < .001^**^), HC and FEP (*P* < .001^**^), and ARMS and FEP (*P* = .0179^*^) in overall decision-making bias with respect to the red frame cue per se (this “general bias” being higher in patients, and especially in FEP).

Condition’s effect differed significantly between HC and ARMS (*P* = .0143^*^), and between HC and FEP (*p* < < 0.0001^**^), but not between ARMS and FEP (*P* = .177). Controls were more biased toward the option indicated by the external cue in the Social condition (*P* = .00174^*^), whereas FEP participants were more biased toward it in the Roulette condition (*P* = .0213^*^). Bias in ARMS was unaffected by Condition (*P* = .781).

The significant Group:IndivBL interaction reflected a stronger IndivBL effect in HC than in both ARMS (*P* < .0001^**^) and FEP (*P* < .0001^**^), with the IndivBL effect moreover being weaker in FEP than ARMS (*P* = .0248^*^).

The effect of RFBL depended differently on Condition in HC than in both FEP (*P* < .001^*^) and ARMS (*P* < .001^*^) patients, who did not differ from one another in this respect (*P* = .809). Whereas controls’ sensitivity to the frame’s predictive value was higher in the Roulette than the Social condition (*P* < .0001^**^), there were no such differences in sensitivity to the value of social versus non-social indirect information within FEP (*P* = .699), nor within ARMS (*P* = .979).

Importantly, the significant difference between effects-coded model coefficients Cond_SOCIAL:Group_HC_:IndivBL and Cond_SOCIAL:Group_HC:RFBL confirms that the effect of Condition on controls’ sensitivity to the red frame’s value is attributable to that cue’s (social) source, and not simply an incidental effect of (social) context associated with the Condition manipulation in general. Unlike what we might expect if (for example) controls found the social context particularly distracting, there is no analogous effect of Condition on HCs’ use of direct information from individual learning about outcomes associated with option’s color. Therefore, the Condition manipulation, here as in previous studies using this paradigm,^32^ seems to be working as intended—specifically altering the perceived sociality of the source generating the indirect information assayed by RFBL—to drive the finding that (while patients use indirect information equally adeptly across both Conditions) healthy cognition is associated with reduced sensitivity to fluctuations in the predictive value of social, versus non-social, external cues.

### Simulations

These results demonstrate that how decision-making under uncertainty is influenced by external cues, and how sensitively it incorporates information from ongoing observational learning about their predictive validity, depends on the perceived sociality of their external origin differently across groups. As discussed, the foregoing computational analysis was needed to: (1) objectively assay the information available from each cue, vis-à-vis an ideal Bayesian observer estimating option value based on learning about that cue’s associative relationship with reward; (2) statistically parse out the separable contributions to trialwise choices made by each predictor. The results of the GLM used to statistically parse out the effects of these various predictive factors can be illustrated in simulations: by allowing each of the factors whose values fluctuated during a session to vary across its observed experimental range, while holding the other’s value constant, and examining the resulting impact on model-predicted behavior at each level of the experimental manipulations of interest (Group and Condition). The GLM shows how choices reflect the linear combination of all factors relevant to predicting observed behavior on every actual trial of the experiment: by simulating model-predicted choices as a function of variation in one factor only, that factor’s effect can be visually compared across different levels of Group and Condition.

**Figure 4:**
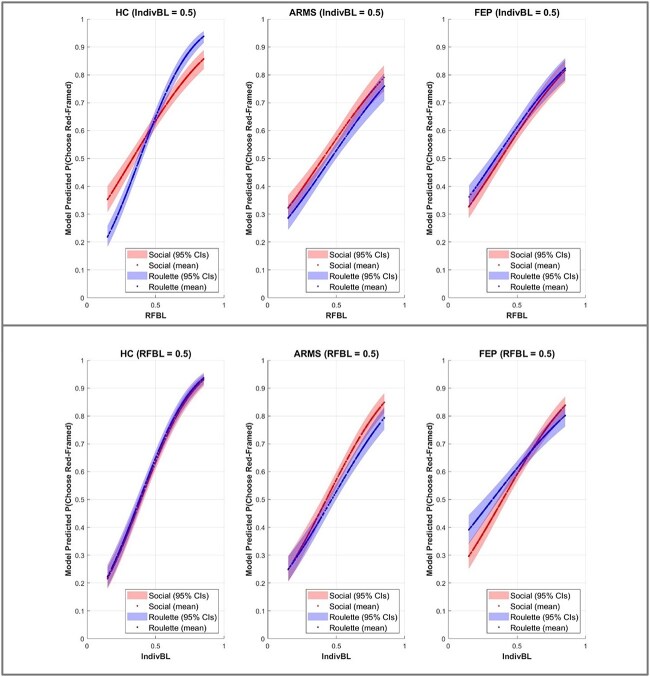
(A) Separate plots for HC, ARMS, and FEP showing how RFBL influences model-predicted probability of choosing the red-framed option in each experimental condition, under the simulated situation wherein the direct cue (option color) is entirely uninformative (ie, IndivBL = 0.5 for all values of RFBL). 95% confidence intervals for model-predicted RF choices are included as shaded areas. (B) Separate plots for HC, ARMS and FEP showing how direct information available from the color of an option, IndivBL, influences model-predicted probability of choosing that option, in each experimental condition (social and roulette), under the simulated situation wherein the indirect cue (framedness) is entirely uninformative, ie, RFBL = 0.5 for all values of IndivBL. 95% confidence intervals for model-predicted choices are included for each condition.

The significant group differences in how perceived (non)sociality of its origin affected decision-making bias toward the external cue, and adaptive use of indirect information about option value available from learning about it, can be seen in simulated data that discounts the additional variance in the probability of choosing or foregoing the red-framed option that, on any given trial of a session, was contributed by direct information about the value of its color (Figure 4A). By altering the predictive significance of an option’s color to be maximally ambiguous on all trials (ie, fixing IndivBL = 0.5), direct information is rendered epistemically useless. Effects of experimental factors of interest on subjects’ sensitivity to indirect information can thereby be visualized, by plotting model-predicted probability of choosing the framed square against RFBL, in isolation from the noise that would be introduced by fluctuations in the value of that square’s color during an actual session.

The key findings from the computational analyses are *illustrated* in Figure 4A (model-predicted probability of choosing the red-framed option, when IndivBL = 0.5, for all values of RFBL observed by the Bayesian-learner throughout actual experimental sessions). Bias and sensitivity to the indirect cue are reflected in the intercept and slope, respectively, of model-predicted choice probability plotted against RFBL (with IndivBL fixed at 0.5). That the blue line is steeper than the red line only in HC reflects controls’ uniquely blunted sensitivity to the indirect cue’s value in the Social compared to the Roulette condition. Furthermore, only in HC does the red line intercept the *y*-axis significantly above the blue line, illustrating the greater bias toward social than non-social external cues characterizing controls specifically.

Figure 4B illustrates how changes to the predictive value of information available from individual experience of outcomes associated with the direct cue (option color) influences model-predicted choice probability when the indirect cue is entirely unhelpful (ie, RFBL is fixed at 0.5). Although learning from direct information was not of primary interest in this experiment, these simulations of IndivBL’s effects accord with the GLM and ANOVA results. Having rendered the external cue entirely uninformative, HC participants’ increased bias toward the external cue in the Social vs Roulette condition is noticeably absent (Figure 4B), suggesting controls’ “social bias” may operate at the level of conflict resolution on trials wherein the 2 independent cues are in competition (ie, when framedness and color pull an option’s value estimates in different directions), although it is also possible that controls’ blunted sensitivity to RFBL in the Social condition (within which their choices are appreciably more sensitive to changes in IndivBL than RFBL) meant their underlying decision bias toward social sources would have more opportunity to manifest when IndivBL provided no useful information, due to greater decision uncertainty caused by the increased overall difficulty in estimating options’ values based on learning about a cue for which their sensitivity was detrimentally affected.

Although this did not emerge as a significant Group difference, a tendency in FEP to more adaptively use direct information (IndivBL) in the Social than the Roulette Condition can be seen in the relative slopes of the lines in Figure 4B’s right-hand subplot. That is, in FEP, choice is more sensitive to changes in option value as estimated by IndivBL in the Social (versus Roulette) condition. This is despite IndivBL reflecting individual learning about the relative value of blue versus green: color being a “direct cue” whose significance in either session is predicated entirely ontrial-and-error learning, without pertaining at all to the Condition manipulation (which alters only the meaning of the other, external cue, ie, alters only the perceived source of the red frame). Thus, perhaps the overarching social context, in the session wherein the external cue was interpreted as indicating other people’s behavior, induced greater attention to and reliance on direct information acquired from personal experience in FEP—an intriguing possibility which, given its non-significance in the ANOVA, will not be interpreted further.

In summary, these simulations illustrate graphically the results of the computational modeling analysis. By holding constant the information available through learning from individual experience of blue and green options’ outcome histories (IndivBL), we saw how changes in the value of the external cue (RFBL) systematically influence choice probability. It can be seen from the blue and red lines’ respective slopes that RFBL influences behavior more adaptively in the non-social (Roulette) than the Social condition, in HC only. In contrast, in ARMS and FEP, RFBL’s influence is comparable between Social and Non-Social conditions (see the extensive overlap between 95% CIs around the lines showing RFBL’s influence in these groups). This illustrates the significant 3-way interaction wherein only controls showed diminished sensitivity to external information as a function of its sociality. This blunted sensitivity, which is not present in ARMS or FEP, is detrimental to controls’ conformity with Bayesian optimality in the social condition. That this effect is unique to the external cue and the indirect information available from it (RFBL), rather than reflecting a more general impact of the Social condition on controls’ learning and decision-making (one that would extend to their use of asocial information available from personal observation of color’s reward history) is evident from Figure 4B which shows how IndivBL (when its effect isolated by holding RFBL constant) influences controls’ decision-making equivalently across both Conditions.

Likewise, in Figure 4A the distinctly lower intercept of the blue (Non-Social) than red (Social) line, evident only in HC, illustrates that when all information learnt about an external cue (RFBL) suggests it predicts non-reward, controls’ adaptive avoidance of it is selectively impaired in the social condition. This illustrates the greater response bias toward social than non-social cues, which the Group:Condition interaction showed was unique to controls. The effect of Condition on HCs’ bias reflects another sense in which responses to an external cue suffer a departure from optimality when that cue’s source is perceived as social, compared to controls’ more strictly value-based choices during the Roulette condition, wherein the same external cue is perceived to be non-social and controls are less biased by it but more sensitive to learning about it as a result.

## Discussion

We assessed how choices on a simple learning task were influenced by direct information (from individual trial-and-error learning) and indirect information (from external “hints,” generated from either a social or non-social source). Overall tendency toward choosing the externally-cued option was lower in controls than both clinical groups, and higher in FEP than ARMS. Interestingly, this bias toward the external cue was affected by its perceived source differently in controls than patients. HCs showed a relatively greater bias toward the social external cue, while in ARMS bias was unaffected by the cue’s perceived origin and, strikingly, the FEP group showed the opposite effect: a relative “non-social bias.” Furthermore, groups were differentially sensitive to the external cue’s actual utility (ie, to whether the red frame likely indicated the correct option or not), depending on its perceived origin. HCs, though biased toward the social cue, were selectively blunted in their ability to use information from learning about its value compared to when its origin was non-social. That is, they were less likely to use information accrued from past trials about the current predictive value of social (versus non-social) cues.

In short, only HCs were more biased toward the external cue, and less sensitive to indirect information available from learning about it, when it had a social origin (ie, reflected other people’s past decisions). It is striking that perceived social origin of an external cue influenced responses in a biased but non-helpful way among HCs, who could more discerningly use non-social information to optimize performance. While, for the non-social condition, they distinguished between when the external “hint” was currently a helpful indicator of reward (and should guide choices toward the red-framed option) and when it was systematically misleading (and should guide choices toward the unframed option), they were more biased toward the frame, and less sensitive to its current predictive value, when perceiving it as social.

Notably, overall bias toward following external “hints” was higher in both patient groups than controls, and higher in FEP than ARMS. This elevated bias toward the red-frame stimulus is consistent with theoretical suggestions that psychotic experiences are underpinned by enhanced motivational salience of external cues.^40,41^ It also accords with previous observations of faster reaction to irrelevant cues in both FEP and ultra-high risk states, suggesting that aspects of aberrant salience affecting decision-making characterize the earliest emergence of psychosis.^42–44^ A general motivational bias toward external cues, irrespective of their perceived meaning or contextual relevance, could arise from an inflated estimation of environmental uncertainty, with external prompting perhaps being favored as an expedient heuristic strategy for action selection. Such uncertainty-driven elevation of salience could itself promote formation of unwarranted beliefs about neutral stimuli across various learning paradigms.^45–47^ This would be in keeping with our observation that overall response bias toward the externally-cued option in general, while elevated in both patient groups, was especially so in FEP. This difference may reflect the fact that the FEP group were experiencing an established psychotic state, while symptoms in the ARMS group were more subtle and indicative of a prodrome. In this respect it is noteworthy that an implicit “idiosyncratic bias towards…unreliable and therefore subjectively irrelevant cue feature(s)” has been reported to characterize patients with a relatively long-standing schizophrenia diagnosis in particular.^48^ Thus, the difference in overall bias between FEP and ARMS would be consistent with the possibility that this general bias toward salient external stimuli worsens as illness progresses.

Strikingly, while HCs were more biased toward cues of social than non-social origin, choices in FEP, as well as being more biased toward external cues generally, were especially biased by those perceived to be non-social. Indeed, the FEP group’s pattern of bias appeared to confer a relative advantage in dealing with social compared to non-social cues. Conversely, HCs were more likely to be misled by external cues of social origin, which tended to bias them away from optimal choices based on estimated values derived from individual and observational learning.

Considering the disturbances to social cognition and functioning in psychosis, why was the generally pronounced tendency toward behavioral capture of responses by a salient external cue in FEP ameliorated when that cue was social? This seems at odds with a domain-specific account under which social cues are more susceptible to aberrant motivational salience attributions. The group differences in Condition’s effect on bias may reflect subjects’ different pre-existing beliefs about the typical reliability of external information from social versus non-social sources. Although detrimental to controls’ behavioral optimality in the Social condition, a greater decision bias toward social external cues could be the hallmark of “dispositional affective trust”: a high-level prior expectation that in general, other people are a particularly valuable source of information.^12^ Controls’ “social bias,” though suboptimal relative to Bayesian standards on this task, may often be advantageous in the real world.^12,32,49^ Underestimation of social cues’ typical epistemic value, in patients, might manifest in tendencies to generate beliefs whose content is less constrained by shared cultural schemas, and to evaluate beliefs relative to eccentric or socially non-normative standards of evidencing and justification. Both tendencies would confer a predisposition to forming delusional world-models.^50–52^ Further, evidence suggests the developmental impact of affective trust deficits results in frequent negatively-valenced and unpredictable social experiences,^53–55^ which can provide an impetus for persecutory interpretations.^56^ While speculative, this could explain the pattern observed in how social and non-social cues were taken into account as a basis for decision-making in FEP.

As shown, HCs’ elevated bias toward social hints was accompanied with a reduced capacity to track their objective value (RFBL) as compared to non-social hints’. Perhaps HCs, through enhanced mentalizing abilities,^57^ adjudge that human decisions, though useful, are more fallible or imprecise than those of a computer, which could be more precisely programmed as either reliably helpful or reliably misleading (across different phases of the session). This could confer an overall tendency to include the social hint in decision-making, but to be relatively insensitive to fluctuations in how helpful it is currently. While the experimental design does not allow us to test this possibility, the overall results are compatible with it, and with recent theoretical work demonstrating that delusions can result from inappropriately high expectations of precision in one’s model of inherently-noisy agents.^58^

Studies of social or non-social inference in psychosis using similar probabilistic reversal learning tasks are admirably reviewed elsewhere.^59^ One study found patients with schizophrenia and borderline personality disorder made greater use of information from learning about an intrinsically-social external cue’s implicit predictive relationship with option outcomes.^23^ Though this finding is consistent with ours, important methodological differences preclude one-to-one comparison: that study lacked a non-social control condition, and information’s sociality was not dissociated from its objective uncertainty. However, at the stimulus level, “gaze direction”^23^ represents a more ecologically valid “social cue” than the abstract red frame used here to manipulate the external cue’s semantic meaning independently from its perceptual properties. Calls for tighter non-social control^20,26,29,60,61^ and greater ecological validity^60,62^ in social learning studies might be simultaneously satisfied by combining these distinct approaches.

Our sample size is a significant limitation: replication in a larger sample would be warranted to corroborate our findings. Having demonstrated this task’s feasibility in patients, future larger-scale clinical studies deploying it could bring greater empirical clarity to the debate between domain-specific and domain-general accounts.^29–31^ For example, the possibility that persecutory delusions in particular are associated with reduced “social bias” could, in a larger sample, be tested this way. The novel methodology, of assaying passive observational social learning (absent any competitive or interpersonally-deceptive context) in direct relation to a non-social control condition, allowed us to provide some direct empirical support for the domain-general account,^29^ though our sample size constitutes a limitation.

In summary, bias toward a perceptually-identical cue as a function of its perceived social or non-social origin differed across groups: controls’ relative “social bias” was absent in ARMS, and directionally-reversed in FEP. Further, only controls (not patients) showed reduced sensitivity to information available from learning about social, compared to non-social, external cues. While these findings indicate fundamental differences in how people with, or at risk for, psychosis process information from social versus non-social sources, they are inconsistent with any simple “social deficit” account attributing psychotic symptoms’ characteristic content to domain-specific impairments particularly affecting social information-processing.

## Supplementary Material

sgag005_Supplementary_materials

## Data Availability

Data are available from the authors upon reasonable request. Currently (as of 20/06/2025) NSPN data can be used only to verify and validate findings in NSPN publications (https://nspn.org.uk/nspn-data/).
