# Supplementary material for: Differential Impact of Social and Non-social Cues on Decision-making Across At-risk Mental State, Early Psychosis, and Healthy Participants
Source: Schizophr Bull Open. 2026 Feb 26;7(1):sgag005. doi: 10.1093/schizbullopen/sgag005 (PMC13191111; doi:10.1093/schizbullopen/sgag005)
Supplement: sgag005_Supplementary_materials [file sgag005_supplementary_materials.doc]

**Supplementary Information**

Participant instruction scripts:

Social Condition:
“On each trial, in the following experiment, you will see a blue and a green box. Your task is to pick the box most likely to give you reward. Things go in phases in this task so sometimes you may be in a blue phase where the blue box will lead to reward, whereas other times you may be in a green phase. Before you make your choice you will see the most popular choice selected by a group of four participants (2 males and 2 females) who previously played the same task. The only catch is that their responses have been juggled. So in some phases they won’t seem very useful – for example they could be guesses from the very beginning of the task when they had little experience. In other phases, however, they will seem quite useful – for example responses from later in the task when they had had the opportunity to practice a bit more.”

Non-Social (Roulette) Condition:
“On each trial, in the following experiment, you will see a blue and a green box. Your task is to pick the box most likely to give you reward. Things go in phases in this task so sometimes you may be in a blue phase where the blue box will lead to reward, whereas other times you may be in a green phase. Before you make your choice you will see a computer-generated suggestion. The computer has generated this suggestion using virtual roulette wheels. On each trial the computer spins the roulette, if the ball lands on black the computer will put a frame around the correct answer, if the ball lands on red the computer will frame the incorrect answer. The only catch is that there are different types of roulette wheel. Some roulette wheels are half red and half black. This type of roulette is equally likely to give you correct and incorrect suggestions. However, others are biased. This type of roulette will give you either mostly correct or mostly incorrect suggestions. Once the computer has selected a roulette wheel it will stick with that wheel for a while. However, it will switch between the various different roulette wheels throughout the course of the experiment.”

Sample Demographic and Clinical Characteristics:
Table S1: Sex and Age Characteristics of HC, ARMS and FEP groups.

|  | **HC** | **ARMS** | **FEP** |
| --- | --- | --- | --- |
| **Sex** | 8 Female; 6 Male | 3 Female; 9 Male | 3 Female; 11 Male |
| **Age (years)** | Mean = 22.86  s.d.= 3.46  Range = 18-27 | Mean = 22.08  s.d. = 4.21  Range = 17-33 | Mean = 24.21  s.d. = 5.21  Range = 17-35 |
| **Age (years): Male** | Mean M = 20.83  s.d. M = 3.817  Range M = 18-27 | Mean M = 22.33  s.d. M= 4.74  Range M = 17-33 | Mean M = 22.73  s.d. M = 4.37  Range M = 17-28 |
| **Age (years): Female** | Mean F = 24.38  s.d. F = 2.39  Range F = 21-27 | Mean F = 21.33  s.d. F = 2.52  Range F = 19-24 | Mean F = 29.67  s.d. F = 4.73  Range F = 26-35 |

Table S2: Clinical Characteristics of HC, ARMS, and FEP groups. For numerical measures the group mean (μ) is indicated as the first number, with the group standard deviation (s.d.) being indicated beside it in brackets: μ (s.d.)

|  | **HC** | | **ARMS** | | **FEP** | |
| --- | --- | --- | --- | --- | --- | --- |
| **IQ** | 118.214 (9.325) | | 115.00 (13.027) | | 109.273 (14.437) | |
| **CAARMS Non-Bizarre Ideas** | 1 (0) | | 3.900 (1.290) | | 5 (2) | |
| **CAARMS Unusual Thought Content** | 1.33 (0.651) | | 3.900 (1.912) | | 5.455 (1.508) | |
| **CAARMS Perceptual Abnormalities** | 1.5 (1) | | 3.400 (1.430) | | 5.181 (1.662) | |
| **PANSS P1 Delusions** | 1.111 (0.333) | | 2.143 (0.69) | | 5.20 (1.789) | |
| **PANSS P2 Conceptual Disorganization** | 1 (0) | | 1.429 (0.787) | | 2.20 (1.095) | |
| **PANSS P3 Hallucinations** | 1.222 (0.667) | | 2.143 (1.492) | | 3.400 (1.140) | |
| **PANSS Total POSITIVE** | 7.462 (1.127) | | 12.93 (1.492) | | 18.750 (6.017) | |
| **PANSS Total NEGATIVE** | 7.539 (1.392) | | 13.571 (5.840) | | 12.583 (7.96) | |
| **Antipsychotics** | Yes=0 | No=14 | Yes=1 | No =11 | Yes =11 | No=3 |
| **Other Psychiatric Medicines** | Yes=1 | No=13 | Yes=6 | No=6 | Yes=4 | No=10 |

Computational Model Fit Statistics:

Table S3: Model fit statistics for Winning Model (fixed-effects only, as described by Equation 1 in main manuscript) and Alternate Models 1 - 5 (fixed-effects as described by Equation 1, plus various random effects of subject as specified below). Note that lower AIC, lower BIC, higher LL (i.e. LL less far below zero), and lower deviance values are indicative of better model fit.

|  | **Random Effect Terms (Formula)** | **AIC** | **BIC** | **LL** | **Deviance** |
| --- | --- | --- | --- | --- | --- |
| **Winning Model*** | **No random effects** | **33465*** | **33594*** | **-16714*** | **33429*** |
| Alternate Model 1  *(random intercepts)* | + (1 | subID) | 33860 | 33997 | -16911 | 33822 |
| Alternate Model 2  *(random intercepts and slopes; all random effects uncorrelated with one another)* | +(1 | subID)  + (IndivBL | subID)  + (RFBL | subID) | 35322 | 35473 | -17640 | 35280 |
| Alternate Model 3  *(random intercepts and slopes; random intercepts correlated with random effects of IndivBL and RFBL, random slopes uncorrelated with one another)* | + (1 + IndivBL | subID)  + (1 + RFBL | subID) | 35407 | 35579 | -17679 | 35359 |
| Alternate Model 4  *(random intercepts and random slopes; random slopes of IndivBL and RFBL correlated with one another; both random slope terms uncorrelated with random intercept term)* | + (1 | subID)  + (IndivBL + RFBL | subID) | 35305 | 35463 | -17630 | 35261 |
| Alternate Model 5  *(random intercepts and random slopes for IndivBL and RFBL; all random effects correlated with one another)* | + (1 + IndivBL + RFBL | subID) | 35403 | 35575 | -17677 | 35355 |
